# Rapid sexual and genomic isolation in sympatric *Drosophila* without reproductive character displacement

**DOI:** 10.1002/ece3.3893

**Published:** 2018-02-11

**Authors:** Roman Yukilevich, Luana S. Maroja, Kim Nguyen, Syed Hussain, Preethi Kumaran

**Affiliations:** ^1^ Department of Biology Union College Schenectady NY USA; ^2^ Department of Biology Williams College Williamstown MA USA

**Keywords:** gene flow, genomic divergence, isolation by distance, population structure, range expansions, reproductive character displacement, secondary contact, sexual isolation

## Abstract

The rapid evolution of sexual isolation in sympatry has long been associated with reinforcement (i.e., selection to avoid maladaptive hybridization). However, there are many species pairs in sympatry that have evolved rapid sexual isolation without known costs to hybridization. A major unresolved question is what evolutionary processes are involved in driving rapid speciation in such cases. Here, we focus on one such system; the *Drosophila athabasca* species complex, which is composed of three partially sympatric and interfertile semispecies: WN, EA, and EB. To study speciation in this species complex, we assayed sexual and genomic isolation within and between these semispecies in both sympatric and allopatric populations. First, we found no evidence of reproductive character displacement (RCD) in sympatric zones compared to distant allopatry. Instead, semispecies were virtually completely sexually isolated from each other across their entire ranges. Moreover, using spatial approaches and coalescent demographic simulations, we detected either zero or only weak heterospecific gene flow in sympatry. In contrast, within each semispecies we found only random mating and little population genetic structure, except between highly geographically distant populations. Finally, we determined that speciation in this system is at least an order of magnitude older than previously assumed, with WN diverging first, around 200K years ago, and EA and EB diverging 100K years ago. In total, these results suggest that these semispecies should be given full species status and we adopt new nomenclature: WN—*D. athabasca*, EA—*D. mahican*, and EB—*D. lenape*. While the lack of RCD in sympatry and interfertility do not support reinforcement, we discuss what additional evidence is needed to further decipher the mechanisms that caused rapid speciation in this species complex.

## 1 INTRODUCTION

A major goal in evolutionary biology is to understand the factors that drive speciation. This includes the study of genetic and phenotypic changes that underlie reproductive isolation and assessing whether genetic isolation has been attained in sympatry (Coyne & Orr, 2004; Mayr, 1963). Some recent notable cases where the genetic and phenotypic changes causing speciation have been deciphered include: adaptive radiation of cichlids (Barluenga, Stölting, Salzburger, Muschick, & Meyer, 2006; Seehausen & van Alphen, 1999; Seehausen et al., 2008; Wagner, Harmon, & Seehausen, 2012), divergence of the benthic and limnetic morphospecies of sticklebacks (Rundle, Nagel, Boughman, & Schluter, 2000; Schluter, 2000), rapid divergence of Hawaiian crickets (Mendelson & Shaw, 2005), and the incipient speciation of *Rhagoletis* host races (Bush, 1969; Feder, Chilcote, & Bush, 1988). These and other similar cases of speciation exhibit strong sexual isolation due to rapid divergence in mating preferences and sexual cues.

The importance of sexual isolation for speciation is well known and is supported by the pattern of enhanced sexual isolation in sympatric *Drosophila* species (Dobzhansky, Ehrman, & Kastritsis, 1968; generalized by Coyne & Orr, 1989, 1997). By revealing that sexual isolation evolves much more rapidly in sympatric relative to allopatric taxa, Coyne and Orr (1989, 1997) emphasized that speciation may work differently in sympatry versus allopatry and that processes such as reinforcement in sympatry may be important. This is because reinforcement (i.e., selection to avoid maladaptive hybridization) explicitly predicts that sexual isolation should evolve faster in sympatry than in allopatry (Coyne & Orr, 1989, 1997, 2004; Dobzhansky et al., 1968; Noor, 1997; Servedio & Noor, 2003). Further evidence that reinforcement has contributed to speciation across sympatric *Drosophila* is supported by a general relationship between strength of sexual isolation and cost of hybridization both across these species and between reciprocal crosses within species (Yukilevich, 2012).

However, rapid sexual isolation in sympatry can only be explained by reinforcement if species pairs show costs to hybridization. Interestingly, as noted by Yukilevich (2012) and Turelli, Lipkowitz, and Brandvain (2014), roughly a quarter of sympatric *Drosophila* species that show high rates of sexual isolation appear to be interfertile (i.e., producing fertile and viable hybrids) and thus do not show obvious costs to hybridization. While it is still unknown whether there are other costs to hybridization, these results potentially suggest that other processes (e.g., sexual and/or ecological divergent selection) may also contribute to enhanced sexual isolation in sympatry (e.g., Yukilevich, 2012; Turelli et al., 2014). So far, very little attention has been given to understanding speciation between interfertile sympatric *Drosophila*.

**Figure 1:**
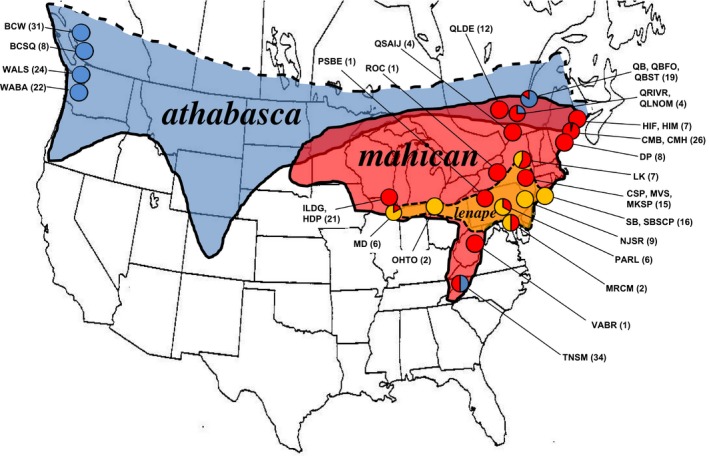
Geographical range map of the *Drosophila athabasca* species complex: *D. athabasca* (WN, blue), *D. mahican* (EA, red), and *D. lenape* (EB, orange) with specific locations shown as pie charts. Each pie chart represents the relative frequency of the three species in each location based on isofemale lines genotyped and/or phenotyped for species identity (sample size of identified lines used per location is shown in parentheses). See Materials and Methods for identification procedure. See Table S1 for detailed location information and description of all lines studied

To shed more light on this question, we need to focus on interfertile sympatric species pairs and test whether reinforcement or other processes are responsible for speciation in such cases. A great system to test this question is the young *Drosophila athabasca* species complex (*obscura* group: *affinis* subgroup; Sturtevant & Dobzhansky, 1936), which contains three widespread semispecies across North America: West Northern (WN), Eastern A (EA), and Eastern B (EB) (see Figure 1 and Miller, 1958a for known ranges). Since the work of D. Miller, these semispecies are known to be interfertile with no apparent evidence of hybrid sterility or inviability (Miller & Westphal, 1967; Hey, 1988; R. Yukilevich; unpublished data).

Despite interfertility, these semispecies show significant sexual isolation, historically assessed based on no‐choice insemination crosses that typically lasted for 10–30 days of confinement (Hey, 1988; Miller, 1958b; Miller & Westphal, 1967; Yoon, 1991). This work showed that the partially sympatric pairs WN–EA and EA–EB had the highest levels of sexual isolation, while the allopatric pair WN–EB had weaker sexual isolation, a pattern that mirrors the more general pattern found across *Drosophila* (Coyne & Orr, 1989, 1997). Recently, Yukilevich, Harvey, Nguyen, Kehlbeck, and Park (2016) used multiple‐choice behavioral mating assays across several isofemale lines to strengthen the result that WN and EA have evolved near complete sexual isolation. However, other taxa comparisons have not been studied using behavioral mating assays.

The three taxa have also diverged in: male copulation duration (Patty, 1975), male courtship song (Chang & Miller, 1978; Miller, Goldstein, & Patty, 1975; Yoon, 1991; Yukilevich et al., 2016), female mating preferences for conspecifics (Yukilevich et al., 2016), cuticular hydrocarbons (Yukilevich et al., 2016), sperm size (Sanger & Miller, 1973), and body size and pigmentation (Sturtevant & Dobzhansky, 1936). However, Yukilevich et al. (2016) recently demonstrated, using playback song experiments with wingless males, that sexual isolation (between WN and EA) is exclusively due to divergent female mate preferences for conspecific male song and that cuticular hydrocarbons play no role in sexual isolation.

At the genetic level, the *D. athabasca* species complex is known to harbor multiple fixed inversion differences across chromosomes and as many as 70 or more polymorphic inversions (Miller, 1977; Miller & Voelker, 1969a, 1969b 1969c, 1972; Novitski, 1946; Sturtevant & Dobzhansky, 1936). Despite this chromosomal divergence, these taxa show very low levels of allozyme and nucleotide sequence divergence (Ford & Aquadro, 1996; Ford, Yoon, & Aquadro, 1994; Johnson, 1978, 1985; Wong‐Miller, Bracewell, Eisen, & Bachtrog, 2017; Yoon & Aquadro, 1994). Based on these data, Ford and Aquadro (1996) and more recently Wong‐Miller et al. (2017), estimated that the first divergence occurred between WN and eastern taxa only 23,000–35,000 years ago, and then EA and EB diverged only 5,000–9,000 years ago.

Many questions still remain about this system. First, we only have a very rough understanding of sexual isolation in this species complex because much of it is based on unrealistic 10‐ to 30‐day no‐choice insemination crosses that do not accurately reflect how flies interact in nature. Moreover, we do not know whether there is a pattern of reproductive character displacement (“RCD”) in sympatric populations relative to allopatric populations between species pairs that have partial zones of overlap (WN–EA and EA–EB). Finding evidence for RCD would clearly support reinforcement as a driver of speciation and would imply that there are unknown costs to hybridization despite apparent interfertility. Alternatively, failure to find evidence of RCD would suggest that other processes are likely involved (Coyne & Orr, 2004).

Second, we know almost nothing about whether the widely ranging conspecific populations within each group are themselves undergoing sexual and genetic divergence and whether there is any population genetic structure. Such data are necessary to determine conspecific gene flow and dispersibility, which would provide insight about historical opportunities for allopatric isolation in this species complex (Coyne & Orr, 2004).

Third, it is still unclear whether these taxa exchange heterospecific gene flow in sympatry, which is another key criterion for secondary contact and reinforcement scenario (Butlin, 1995; Howard, 1993; Servedio & Noor, 2003). Prior data using allozymes (Johnson, 1978) and chromosomal inversions (Miller & Voelker, 1969a, 1969b, 1972) failed to find any hybrids between WN and EA. More recently, Wong‐Miller et al. (2017) using nucleotide sequences found mixed results, with some analyses showing weak heterospecific gene flow and others failing to support gene flow. However, only several and mostly allopatric isofemale lines were studied, and thus, further work is needed to resolve this question in sympatry.

Finally, there is still a question about the age of this speciation event. Prior estimates of only several thousand years suggest that speciation occurred extremely rapidly. However, such rapid divergence would be very surprising as these taxa have diverged in multitude of phenotypes, behaviors, and chromosomal inversions.

To resolve these questions, we performed extensive behavioral mating tests within and between these taxa and studied the genomes of hundreds of lines in sympatric and allopatric populations to determine population genetic structure, test for heterospecific gene flow, and estimate times of divergence. Our results all point to a reassessment of the *D*. *athabasca* species complex. We find no evidence of RCD in sympatry and instead discovered that these taxa remain virtually completely sexually isolated in sympatry and allopatry. We also find random mating and rampant conspecific gene flow within each taxon, but only zero or very low heterospecific gene flow between these taxa. Finally, we show that these taxa are substantially older than previously assumed and most likely diverged roughly 100–200 thousand years ago. These results imply that the three *D. athabasca* semispecies should be given full species status and we will thus henceforth use the following names: WN—*D. athabasca*, EA—*D. mahican*, and EB—*D. lenape* (see below for details about nomenclature). Below, we discuss why our results are generally not consistent with reinforcement in this system and how this finding relates to the broader pattern of enhanced sexual isolation across *Drosophila*.

## 2 MATERIALS AND METHODS

### 2.1 Study system and rearing conditions

R. Yukilevich collected wild females of all species in this study, including females of *D. athabasca* species complex, *D. affinis*, and *D. pseudoobscura*, from years 2011 to 2016 using banana/yeast baits. Each female was immediately isolated in the field into a vial with Carolina instant *Drosophila* food to establish isofemale genetic lines (see Figure 1 for collecting locations and Table S1 for collection date and additional information about lines used in the study). All lines were maintained and studied in a room at 18–20°C and 50%–60% humidity with a 14:10 light:dark cycle. Classification of *D. athabasca* (WN), *D. mahican* (EA), and *D. lenape* (EB) lines was based on: (1) copulation duration, (2) male courtship song, (3) geographical location, (4) sexual isolation between lines, (5) high *Fst* indel difference at *Period* locus (Ford et al., 1994), and (6) species‐specific SNP difference at the *Nona* locus. No discrepancies were found between these measures among sampled lines (see Table S1). The species names *“D. athabasca”* and “*D. mahican”* that we adopt in this manuscript were originally given as semispecies names by Sturtevant and Dobzhansky (1936), while the species name “*D. lenape*” is given to EB because it largely overlaps with the historical location of Lenape Native American tribes (Grumet, 2012).

### 2.2 Multiple‐choice and no‐choice mating tests

We studied sexual isolation within and between the three behavioral species using many isofemale lines (see Table S2). This work extends our previous survey of sexual isolation between *D. athabasca* and *D. mahican* (see Yukilevich et al., 2016). We now include mating tests between all three behavioral species pairs and substantially increase the number of populations studied across the whole range of each species, including explicitly allopatric and sympatric populations (see Figure 1). All mating tests used virgins separated with CO_2_ anesthesia and placed in pooled sex‐specific vials for 10–20 days (Miller & Westphal, 1967; Patty, 1975). The mating trial started within the first 5 min of “lights‐on.”

As individuals in this species complex congregate on common food sources in the wild (Carson & Stalker, 1951; R. Yukilevich personal observation), we used multiple‐choice mating tests. We performed a total of 110 multiple‐choice mating tests between species, resulting in 1,529 total copulations, and 64 tests within species for 1,021 total copulations. For each replicate, approximately 20 males and 20 females of each species (80 total individuals per bottle) were placed into a clean, clear Polystyrene bottle (dimensions: 14 cm_Length_ × 7 cm_Width_ × 7 cm_Height_) with instant food. The day before each experiment, flies from different lines were fed green or red colored food for identification (McCormick, Inc.; color has no effect on mating and was alternated between replicates; data not shown). Without anesthesia, females of each type were introduced first into the bottle to habituate for 30 s, followed by males of each type. Copulating pairs were aspirated out during the first 15–30 min until 50% of individuals mated (Casares et al., 1998). Pairs were placed into individual vials for their stomach color to be scored under a microscope.

We also performed a total of ten no‐choice mating experiments to complement our extensive multiple‐choice trials. This approach resulted in 228 total copulations across three species pairs (see Table S2). Each experiment consisted of both homotypic and heterotypic reciprocal crosses performed simultaneously in four parallel bottles. Each bottle contained approximately 20 males and 20 females of a given cross, and copulating pairs were aspirated out for 30 min and then counted to determine percentage of mating.

Estimate of sexual isolation was based on the standard sexual isolation index (SI) of Merrel (1950; Malogolowkin‐Cohen, Simmons, & Levene, 1965: SI = (homotypic % matings − heterotypic % matings)/total % matings). The SI ranges from ‐1 (disassortative mating) to 1 (complete sexual isolation), with 0 equal to random mating. For no‐choice mating tests, the index was based on the percentage of matings out of total number of possible females per cross. The *I*_psi_ of Rolán‐Álvarez and Caballero (2000) gave qualitatively identical results (data not shown). An *X* ^2^ contingency test was used to determine significance (Sokal & Rohlf, 1995).

### 2.3 Genomic fragments and DNA extractions

Because the genome of *D. athabasca* species complex was not available, we relied on *D. melanogaster* genome (BDGP release 6: August 2014; genome.ucsc.edu) to identify conserved regions with *D. pseudoobscura* (November 2004 release) that were then used to develop and test primers in the *D. athabasca* species complex. We targeted genomic fragments of about 500 bp that contained largely conserved flanking regions of about 100 bp from each end of fragment. Each primer pair was tested by amplifying the region using PCR and then Sanger‐sequenced (University of Chicago, IL, Genomic Sequencing Center).

The above approach resulted in 64 amplifiable genetic fragments, distributed across all four chromosomes of *D. athabasca* species complex (see below for details). Four of the DNA fragments were sequenced across our samples using Sanger sequencing, and the rest 60 fragments were sequenced using Illumina HiSeq based on multiplex PCR products (see below). We extracted DNA from one to two female individuals per isofemale line, using single‐fly prep with a DNeasy Qiagen kit following the product's protocol. Fly DNA extraction was from isofemale lines that had only been in the laboratory for two to three generations in large numbers (i.e., not inbred). In total, we sequenced 384 individuals across *D. athabasca* species complex and outgroups *D. pseudoobscura* and *D. affinis* for a total of 319 unique isofemale lines (see Table S1).

### 2.4 Multiplex next‐generation sequencing

The 60 primer pairs designed for multiplex PCR reactions were ordered with Illumina Nextera adaptor sequences (TCGTCGGCAGCGTCAGATGTGTATAAGAGACAG and GTCTCGTGGGCTCGGAGATGTGTATAAGAGACAG, respectively). The 60 loci were amplified by multiplex PCR (Qiagen Multiplex PCR Kit^™^) in three separate mixes of 20 primers each. We pooled the three resulting PCR products for each individual (384 total samples) and added Illumina barcodes (N501‐520/S701‐729) via PCR (OneTaq, New England Labs). Finally, we pooled all individuals in a single mix and cleaned the reaction with 1.6× Agencourt AMPure XL beads (Beckman Coulter, Inc.). We ran 384 individuals per lane (two lanes) on an Illumina HiSeq 2500 with paired‐end sequencing (300 bp × 2 PE on MiSeq) (GENEWIZ).

### 2.5 Population genetic analyses

We analyzed sequenced reads in Geneious R9 (Biomatters). After trimming and filtering paired reads at 5% error rate, we mapped all reads per individual to each known reference gene fragment sequence (see above). This resulted in an average coverage of 1,274 reads per individual (95% range: 1,215–1,333) mapped to each reference fragment sequence. We then generated a consensus sequence for each individual trimmed to the reference sequence. For consensus sequence, we recorded ambiguities (i.e., S, W, Y, R, K, M) at positions with minor allele frequency of at least 25% and a minimum coverage of ten reads (individuals with low coverage were visually inspected and assigned as “missing” if data were not high quality). We then aligned the consensus sequences of all individuals to each known reference gene fragment for further analyses.

Of the total 64 gene fragments (60 Multiplex and 4 Sanger), we successfully sequenced isofemale lines for 52 fragments, for an average fragment size of 444 bp (95% range: 403–485 bp). A total of 21 genes were located on the X chromosome (Muller's elements A and D) and remaining 31 genes on three autosomes (Muller's elements: B, C, and E) (see Table S3 for details). In total, these represent 19 coding fragments, four intergenic fragments, 12 noncoding fragments, and 17 fragments spanning coding and noncoding regions. On average, gene fragments were sequenced for 188 isofemale lines (95% range: 160–211). Average sample sizes per fragment were as follows: four lines of *D. pseudoobscura*, six lines of *D. affinis*, 70 lines of *D. athabasca* (WN; 95% range: 59–82), 81 lines of *D. mahican* (EA; 95% range: 73–89), and 26 lines of *D. lenape* (EB; 95% range: 23–30; see Table S3 for details). While multiple individuals from each line were sequenced, all population genetic analyses used only a single individual per line (312 samples) to ensure independent sampling. Statistical tests, unless noted otherwise, were performed using JMP v.4 software (SAS Inc.). Aligned sequences are available in GenBank under MG860929‐MG865277, MG793688‐MG797444 accession numbers.

#### 2.5.1 Population genetic statistics

We used DNAsp5 software (v.5.10.01; Rozas, Sánchez‐DelBarrio, Messeguer, & Rozas, 2003) to analyze basic population genetic statistics across our samples for each of the 52 gene fragments. This included (1) nucleotide diversity (π), defined as the average number of nucleotide differences per site between two sequences, (2) the average number of nucleotide differences (*k*), (3) haplotype diversity (*Hd*), (4) relative sequence divergence (*Fst*), defined as between‐population sequence diversity relative to within‐population diversity (Weir & Cockerham, 1984), (5) absolute nucleotide sequence divergence (*Dxy*), defined as the average number of nucleotide substitutions per site between populations (Nei, 1987), and (6) Fay and Wu's *H*, defined as excess of high‐frequency‐derived alleles.

We only analyzed gene fragments that had a minimum of 10 isofemale lines per species of *D. athabasca* species complex and a minimum of five lines for *D. affinis*. The *Fst* and *Dxy* measures were used to test for isolation‐by‐distance (IBD) patterns within and between species and for the phylogenetic analyses (see below). Geographical distances (in kilometers) between each pair of local populations were calculated using the software Geographic Distance Matrix Generator v.1.2.3 (Ersts, 2006). We also concatenated all of our 52 sequenced fragments in Geneious R9 to produce 22,667‐bp sequence per individual (including any gaps and indels among individuals). The concatenated file was used for additional analyses described below.

#### 2.5.2 Population genetic structure and ancestry of isofemale lines

We used STRUCTURE (2.3.4; Pritchard, Stephens, & Donnelly, 2000) to identify population genetic clusters and to assign individual ancestry across 281 unique isofemale lines spanning 21 geographical populations of *D. athabasca* species complex. No a priori information was given about species identity of individuals. STRUCTURE was based on 4,690 total variable base pairs per individual. We ran simulations for *K* = 2–6, each replicated 20 times with default software settings and 5,000 burn‐in period and 5,000 MCMC run. For each *K*, we determined the value of Ln *P*(*D*) and delta *K* value (Evanno, Regnaut, & Goudet, 2005). For each individual, we also determined the probability of being assigned to a given *K* cluster for simulations with *K* = 3.

#### 2.5.3 Phylogenetic analyses of populations

To determine phylogenetic relationships, we first used average pairwise *Fst* and *Dxy* distances across all 52 gene fragments across populations and clustered using neighbor‐joining (NJ) method. We also used 22,261 bp of consensus sequences for each population and clustered using maximum likelihood (ML) method. *Drosophila affinis* is an outgroup in all analyses. Consensus tree for ML method was based on bootstrapping across 500 replicate phylogenetic trees. Trees were generated with MEGA7.

#### 2.5.4 Principal component analysis of populations

We used principal component analysis (PCA) to cluster 20 geographical populations based on SNP sites across the *D. athabasca* species complex. First, we identified a total of 4,236 variable sites based on eight outgroup *D. affinis* lines and 277 lines from *D. athabasca* species complex (isolated from 22,667‐bp concatenated sequence in MEGA7). We then eliminated singletons and fixed differences relative to *D. affinis* and calculated the average allele frequency per SNP site for each *D. athabasca* species population. A given SNP site had to be represented by at least five lines per population for inclusion in analysis. This resulted in 1,820 informative SNP sites across the complex that were then subjected to PCA to generate covariances, eigenvectors, and loadings for the first two major principal components, using JMP4 software.

#### 2.5.5 Spatial tests of heterospecific gene flow

To test whether partially sympatric species pairs (*D. athabasca–D. mahican* and *D. mahican–D. lenape*) show evidence of gene flow, we first asked whether genetic divergence is lower between sympatric heterospecific populations compared to increasingly distant allopatric populations. To do this, we determined average *Fst* and *Dxy* of each local population with all heterospecific populations and then plotted this value as a function of the local population's minimum geographical distance from the other species in km (based on Geographic Distance Matrix Generator v.1.2.3). The allopatric pair *D. athabasca*–*D. lenape* was used as a control. We analyzed each relationship using regression analysis.

Similarly, we used ABBA–BABA test of gene flow between *D. athabasca and D. mahican* by sampling individuals from zones of sympatry to test whether they are more genetically similar to the other species than sampled allopatric individuals (Green et al., 2010). Starting with the 4,236 variable sites (see above), we identified biallelic sites that had derived alleles in either *D. athabasca* (WN) or *D. mahican* (EA) relative to outgroup *D. affinis* and were polymorphic between two conspecific sequences being compared (e.g., H1 = *D. mahican* allopatric, H2 = *D. mahican* sympatric, *D. athabasca* (WN), *D. affinis*). We randomly picked two conspecific sequences (H1, H2), and for each sequence, we determined the number of sites that matched the other species allele (ABBA or BABA). We then calculated *D = *(no. of ABBA sites – no. of BABA sites)/(no. of ABBA sites + no. of BABA sites). We then tested whether *D* statistic was significantly different from zero across different comparisons with ANOVA, using JMP4 software.

#### 2.5.6 Coalescent demographic simulations

We used *IMa2* software (Hey, 2010a, 2010b) to estimate time of divergence, effective population sizes of each species, and heterospecific gene flow. Two important requirements of *IMa2* model were satisfied in our study: (1) no population genetic structure within each species and (2) only studying nonrecombining gene fragments (Hey, 2010a, 2010b).

To satisfy the first requirement, we studied two nonoverlapping isofemale line datasets: (1) sympatric lines of *D. athabasca* (WN) and *D. mahican* (EA), and 2) sympatric lines of *D. mahican* (EA) and *D. lenape* (EB) (see Table 1 for lines used/locations). This approach explicitly tested for heterospecific gene flow in sympatric populations that are in direct contact with each other and ix00A0;5 t virtually eliminated population genetic structure within each species (see Figure 5 Results below).

**Table 1:** Results of *IMa2* simulations for estimated time of divergence (*T*) and effective population size (*N*e) for each species pair analyzed and comparison of our results to previous estimates

	Wong‐Miller et al. (2017), estimates^a^	Scenario 1: *D. affinis–athabasca* sp. complex 250 KY divergence time^b^	Scenario 2: *D. affinis–athabasca* sp. complex 1.5 MY divergence time^c^	Scenario 3: *D. affinis–athabasca* sp. complex 3.0 MY divergence time^d^
*u*_per site per year_	5.8 × 10^−8^	6.08 × 10^−8^	1.027 × 10^−8^	5.068 × 10^−9^
*T*_*D. athabasca* (WN)–*D. mahican* (EA)_	**34,519 years** (95%: 595–153,052)	**45,441 years** (95%: 35,866–55,665)	**191,695 years** (95%: 154,898–240,407)	**487,355 years** (95%:403,391–626,077)
*T*_*D. mahican* (EA)*–D. lenape* (EB)_	**8,766 years** (95%: 407–15,665)	**21,495 years** (95%: 14,171–301,089)	**98,125 years** (95%: 62,380–1,325,391)	**281,209 years** (95%: 172,604–3,667,355)
*N*e_*D. athabasca* (WN)_	**321,058** (95%: 76,763–2,035,399)	**483,213** (95%: 403,060–574,650)	**2,087,781** (95%: 1,757,492–2,505,696)	**5,438,248** (95%: 4,715,624–6,723,173)
*N*e_*D. mahican* (EA)_	**568,203** (95%: 33,090–1,447,230)	**509,329** e (95%: 431,690–641,357)	**2,225,217** f (95%: 1,882,339–2,796,567)	**5,940,094** g (95%: 5,050,607–7,503,625)
*N*e_*D. lenape* (EB)_	**139,740** (95%: 11,323–150,514)	**149,190** (95%: 110,409–235,687)	**676,331** (95%: 481,427–1,027,686)	**1,959,978** (95%: 1,291,742–2,757,441)

Estimated time of divergence is based on peak probability from IMa2 model with different scenarios (95% confidence intervals shown below each estimate). Note that the IMa2 program calculates the per locus mutation rate based on calculated sequence divergence per locus and a given time of divergence with an outgroup species (i.e., *D. affinis*; see Materials and Methods).

a Values shown are averages of Wong‐Miller et al. (2017) independent estimates for autosomes and X chromosome data (Wong‐Miller et al., 2017 assumed: 5.8 × 10^−9^/gen. and 10 gen./year, so that *u* = 5.8 × 10^−8^ per site per year).

b Assuming a hypothetical (and not previously supported) divergence time of 250,000 years between *D. affinis* and *athabasca* species complex in order to approach divergence time estimates close to estimates of Ford and Aquadro (1996) and Wong‐Miller et al. (2017).

c Assuming divergence time of 1.5 million years between *D. affinis* and *athabasca* species complex based on Carson's (1976) calibration using Hawaiian *Drosophila* divergence and geological island formation time with the estimated Nei's *D* divergence rate of 0.00000025/generation (Nei's *D* of 1 per 2 million years, with two generations per year in Hawaiian slow‐breeding flies).

d Assuming divergence time of 3 million years between *D. affinis* and *athabasca* species complex, which is slightly less than average between two prior published nucleotide sequence‐based estimates: Beckenbach et al. (1993) (2.7 MY) and Russo et al. (2013) (3.6 MY).

e Estimated *N*e is the average between two simulations with different isofemale lines used for *D. mahican* that are sympatric with *D. athabasca* or with *D. lenape*, respectively: 812,902 (*D. mahican–D. athabasca*)/205,755 (*D. mahican–D. lenape*).

f Estimated *N*e is the average between two simulations with different isofemale lines used for *D. mahican* that are sympatric with *D. athabasca* or with *D. lenape*, respectively: 2,867,411(*D. mahican–D. athabasca*)/1,583,023 (*D. mahican–D. lenape*).

g Estimated *N*e is the average between two simulations with different isofemale lines used for *D. mahican* that are sympatric with *D. athabasca* or with *D. lenape*, respectively: 9,207,490 (*D. mahican–D. athabasca*)/2,672,698 (*D. mahican–D. lenape*).

To satisfy the second requirement, we explicitly used only the longest nonrecombining region for each locus, identified with the *IMgc* software package (Woerner, Cox, & Hammer, 2007). For both analyses, we assumed infinite sites model (*I*), a population size prior of 7 (*4N*μ), time since speciation of 4 (*t*μ), and migration rate of 0.5 (m/μ) following an exponential distribution (‐j7 command line).

We calibrated mutation priors for each locus per generation based on previous estimates of divergence time between *D. affinis* and *D. athabasca* species complex (Beckenbach, Wei, & Liu, 1993; Russo, Mello, Frazão, & Voloch, 2013; Tamura, Subramanian, & Kumar, 2004). We assumed 10 generations per year, based on a 5‐ to 6‐month breeding season (Spiess, 1949) and 15 days per generation in the wild. Each dataset contained 50 genes with an average 332 bp per gene, for an average of 55.4 *D. mahican* and 36.5 *D. athabasca* (WN) individuals (*D. athabasca–D. mahican* dataset) and 8.6 *D. mahican* and 16.6 *D. lenape* individuals (*D. mahican*–*D. lenape* dataset). For both datasets, the parameters reached stationarity (no perceivable trends in “trendplots”) after 100,000 generations with a geometric heating scheme of 100 parallel hot chains (hn = 100, ha = 0.99, hb = 0.75). After 110,000 generations of burn‐in, we collected 30,000 genealogies in several separate runs (combined in the L mode) for each dataset.

## 3 RESULTS

### 3.1 Novel geographical sampling extends the ranges of species

Our survey extends knowledge of the distribution of these species (Miller, 1958a; Miller & Jaenike, 1972; Sturtevant & Dobzhansky, 1936). *Drosophila athabasca* (WN) is widely distributed from Maine, United States and Quebec to British Columbia and Alaska (Figure 1). Its northern limit is largely unknown, but appears abrupt at least in Northeastern Canada (R. Yukilevich personal observation). The *D. mahican* (EA) is found from Appalachian range into northeastern United States and extends into northern parts of Midwest. These two species are partially sympatric and syntopic (i.e., found in same traps) across the United States–Canada border from Midwest to Northeast (see Figure 1 and Miller & Jaenike, 1972). In addition, we recently discovered *D. athabasca* (WN) in the Smoky Mountain National Park at highest altitudes (1,300–1,750 km), which are syntopic with *D. mahican*. This is the most geographically isolated and southern known *D. athabasca* (WN) population (Figure 1).

The *D. lenape* (EB) is found mostly in Long Island (NY), New Jersey, and other Mid‐Atlantic states (Figure 1). However, *D. lenape* was collected as far north as upstate NY and as far west as southern Chicago, IL (Figure 1). Its range is largely encompassed within the range of *D. mahican*, but it was found alone in Long Island and New Jersey, suggesting that these two species have some geographical nonoverlap or microhabitat differences. *D. athabasca* and *D. lenape* have never been found in the same location and are almost certainly allopatric.

### 3.2 Sexual isolation between species is virtually complete and shows no evidence of reproductive character displacement

**Figure 2:**
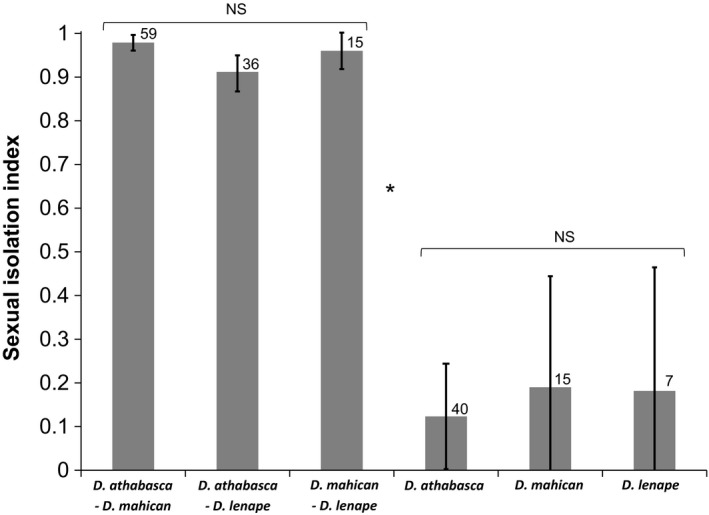
Sexual isolation between and within species of *Drosophila athabasca* (WN), *D. mahican* (EA), and *D. lenape* (EB). The mean SI is shown with error bars indicating 95% confidence intervals across replicates. Number of total replicate tests per comparison shown next to each bar. Results are based mostly on multiple‐choice mating tests across multiple isofemale lines with few no‐choice tests (see Table S2 and text). See Materials and Methods on how mating tests were performed. See Table S2 for detailed description of lines used, specific matings, mating method used, and conditions for each replicate. ANOVA test: NS = not significantly different. Asterisk indicates significance with a post hoc Tukey test

Both pairwise multiple‐choice mating tests as well as no‐choice tests revealed extremely strong levels of sexual isolation between all three species pairs in the *D. athabasca* complex (Figure 2). Average SI was 0.98 between *D. athabasca* and *D. mahican*, 0.90 between *D. athabasca* and *D. lenape*, and 0.97 between *D. mahican* and *D. lenape*, all significantly different from zero (Figure 2). Interestingly, sexual isolation between the allopatric pair *D. athabasca* and *D. lenape* was much higher compared to previous estimates and was not significantly lower than for the two sympatric species pairs (Figure 2).

**Figure 3:**
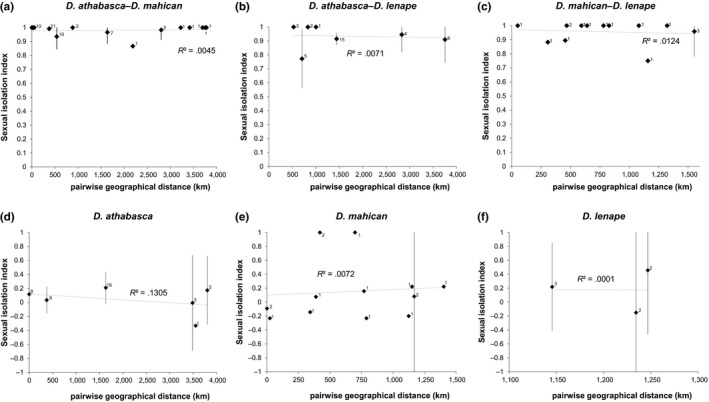
Sexual isolation between (panels: a–c) and within (panels: d–f) species: *Drosophila athabasca* (WN), *D. mahican* (EA), and *D. lenape* (EB). Sexual isolation indexes (SIs) between pairwise comparisons are plotted as a function of geographical distance (km). For multiple comparisons made between the same pairwise localities, the mean SI is shown with error bars indicating 95% confidence intervals across replicates. The number of replicate tests per comparison is shown next to each data point. *R* ^2^ are shown for each plot (none of the relationships are significant). Results are based mostly on multiple‐choice and few no‐choice mating tests across multiple isofemale lines (see Table S2 and text)

We also found no relationship between sexual isolation and geographical distance between heterospecific populations of each species pair (Figure 3a–c). SIs were equally strong between sympatric and allopatric heterospecific populations for partially sympatric species pairs (Figure 3a and c). For instance, sexual isolation was complete between *D. athabasca* and *D. mahican* populations that are nearly 4,000 kilometers apart (Figure 3a). These results unequivocally rule out RCD in sympatry within the *D. athabasca* species complex.

### 3.3 Random mating and near genetic panmixia with weak or no IBD within all three species

In contrast to above between‐species patterns, our within‐species mate choice tests did not show significant deviation from random mating within any species (Figure 2). We also did not find any relationship between sexual isolation and geographical distance between conspecific populations within any taxon, even between *D. athabasca* (WN) populations that are thousands of kilometers apart (Figure 3d–f). This indicates that conspecific populations have not diverged in their mating preferences or in sexual cue traits within any of these species.

The above random mating results reflect near genetic panmixia within each species. First, consistent with previous work (Ford & Aquadro, 1996; Wong‐Miller et al., 2017), all three species showed a significant reduction in nucleotide sequence diversity per site (π) and average haplotype diversity (*Hd*) on the X chromosome compared to autosomes (Figure S1a). On average, *D. lenape* had the lowest nucleotide diversity (π) relative to *D. athabasca* and *D. mahican* (Figure S1b). However, within each species, conspecific populations did not significantly differ from each other in either π or *Hd* (Figure S1b and c). Also, average Fay and Wu's *H* (excess of high‐frequency‐derived alleles) was not significant within any species (Figure S1d).

**Figure 4:**
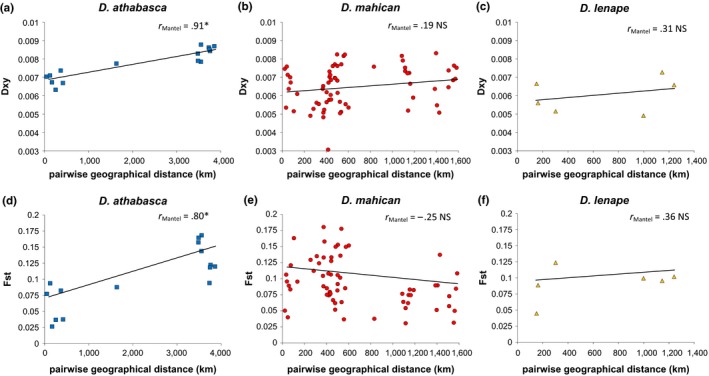
Relationship between pairwise geographical distance (km) of conspecific populations (*x*‐axis) and average measures of sequence divergence (*y*‐axis) within each species: *Drosophila athabasca* (WN, left panels, blue squares), *D. mahican* (EA, center panels, red circles), and *D. lenape* (EB, right panels, orange triangles). Top panels (a–c) show *Dxy* (absolute measure of sequence divergence), and bottom panels (d–f) show *Fst* (relative measure of sequence divergence). Both measures of genetic divergence are averaged across all gene fragments. Only populations with greater than six chromosomes are considered. Mantel tests were performed to determine the significance of each relationship with 1,000 permutation tests of matrix correlations between genetic divergence (*Dxy* or *Fst*) and geographical distance. Significant positive correlations reveal evidence of isolation by distance. Trendlines are shown only to help show patterns. Note the scale of *y*‐axis

Second, we studied both *Fst* and *Dxy* across conspecific populations (values were not different between coding, noncoding, and intergenic genomic fragments; average pairwise values provided in Table S4). Strikingly, we found only significant IBD in *Fst* and *Dxy* between *D. athabasca* (WN) populations that were separated by nearly 4,000 km (Figure 4). At relatively smaller geographical scales (less than 2,000 km apart), there is no significant relationship between genetic divergence and geographical distance within any species, thus failing to show any evidence of IBD within *D. mahican* or *D. lenape* (Figure 4).

**Figure 5:**
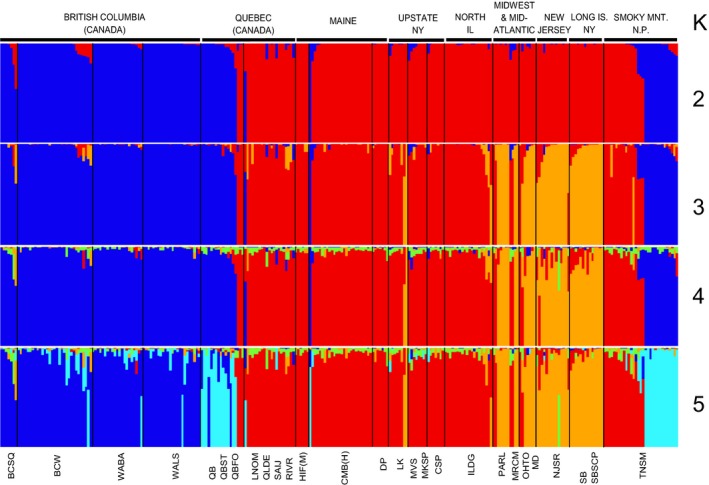
Genetic structure and ancestry of 281 individuals of the *Drosophila athabasca* species complex based on 4,690 total variable base pairs with software STRUCTURE (v 2.3.4). Species designation is as follows: *D. athabasca* (WN, blue/turquoise), *D. mahican* (EA, red), *D. lenape* (EB, orange). *K* = 2–5 plots are shown with default software settings and 5,000 burn‐in period and 5,000 MCMC runs (see Materials and Methods). Specific plot shown is the highest probability run for each *K* among 20 runs per *K* (other runs not shown). On average, *K* = 3 had maximal average value of Ln *P*(*D*) and delta *K* value (Evanno et al., 2005; Table S6). Samples are organized into 21 geographical populations (divided by black lines and abbreviations shown below the plots) and grouped into larger geographical regions across North America (shown above plots and designated with thick black bars)

To further test for population genetic structure across our samples, we analyzed all available SNP sites across all 281 sequenced isofemale lines of *D. athabasca* species complex using STRUCTURE (v.2.3.4; Pritchard et al., 2000). At *K* = 2, genetic separation was evident between *D. athabasca* (WN) versus the two eastern species (*D. mahican* and *D. lenape*), and at *K* = 3, isofemale lines clustered into the three designated species (Figure 5). At *K* = 5, there was an additional split within the species *D. athabasca* (WN): grouping Quebec, Maine, and Smoky Mountain N.P. lines in one group versus British Columbia and Washington lines in another (Figure 5). No further population genetic structure was detected with higher *K* (data not shown).

**Figure 6:**
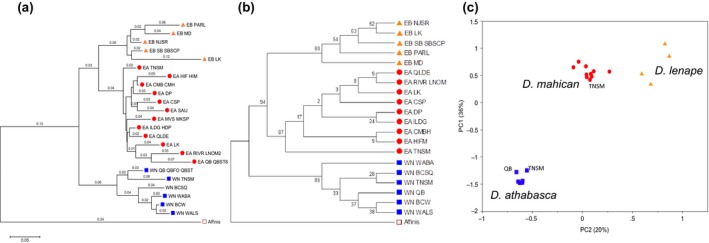
Phylogenetic and principal component clustering analyses of populations from the three species: *Drosophila athabasca* (WN, blue, squares), *D. mahican* (EA, red, circles), and *D. lenape* (EB, orange, triangles/diamonds). *D. affinis* was the outgroup. See text and Figure 1 for locations of abbreviations of specific locations. (a) Phylogeny inferred using the neighbor‐joining (NJ) method based on average pairwise *Fst* values across all 52 gene fragments (see Materials and Methods). Sum of branch length = 1.7 (MEGA7). Minimum number of sequences allowed per population was six chromosomes. Branch lengths are shown. Phylogeny based on *Dxy* distances showed qualitatively similar results (data not shown). (b) A maximum likelihood consensus phylogeny with bootstrap values based on 22,261‐bp consensus sequences across populations (*D. mahican* QB, MVS/MKSP, and SAIJ populations were not included due to small sample sizes). Bootstrap supports (500 replicates) are shown next to the branches. (c) Principal component analysis (PCA) based on covariances across a total of 1,820‐bp SNP sites across populations of each species (same populations as in panel b, except *D. lenape* LK population was not included due to small sample size). PC1 explains 36%, and PC2 explains 20% of total genetic variation across populations. Results were qualitatively similar when different nonoverlapping sets (500 bp per set) were used to generate PCA. Some sympatric locations are labeled

At the population level, we found that all populations clustered according to their species identity, regardless of whether we used a distance‐based phylogeny, a whole‐sequence consensus phylogeny, or a PCA (Figure 6). As expected, the first split was between *D. athabasca* versus the two eastern species, confirming that *D. mahican* and *D. lenape* are sister species (Figure 6). However, at the within‐species level, there was little overall genetic resolution (supported by variable internal nodes between Figure 6a and b and by low bootstrap values for many internal nodes in Figure 6B).

### 3.4 Mixed evidence for heterospecific gene flow between sympatric species

**Figure 7:**
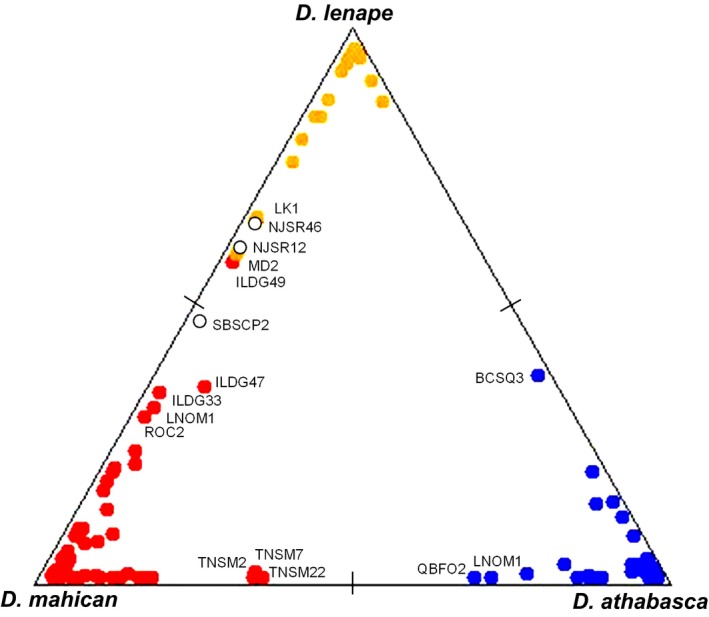
Inferred ancestry (probability of being assigned to a given species) of 281 individuals based on 4,690 total variable base pairs with software STRUCTURE (v 2.3.4) for *K* = 3 run. *Drosophila athabasca* (WN, blue), *D. mahican* (EA, red), and *D. lenape* (EB, orange). Species designation for each isofemale line (represented by a sequenced individual) was established using phenotypic data (male courtship song, copulation duration, and sexual isolation) and geographical information (see Table S1 and text). Open circles represent individuals (lines) for which species designation using phenotypic/geographical data was not determined. Only individual isofemale lines that have a relatively low (70% or less) probability of being assigned to a given species are labeled

First, using STRUCTURE on 281 lines, we found that 265 lines (94%) had very high probability of assignment to one of the three species (above 95% likelihood), with 16 remaining lines (6%) showing lower probability (Figure 7). However, the latter lines all exhibited pure species behavior in male song, copulation duration, and/or sexual isolation (Table 1).

**Figure 8:**
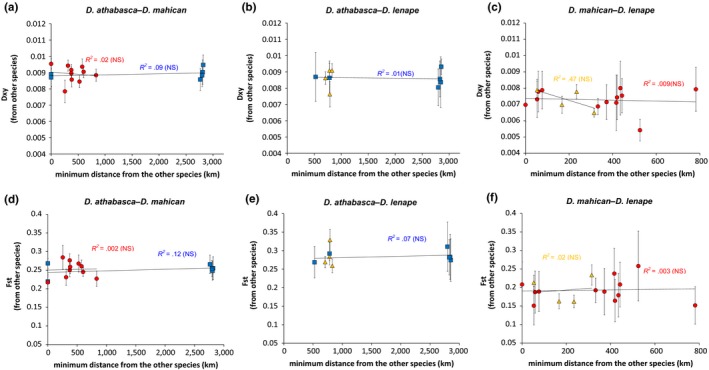
Relationship between the minimum geographical distance (km) of a focal population to the closest population of the other species and its average measure of sequence divergence to all heterospecific populations. Left panels (a and d) compare *Drosophila athabasca* and *D. mahican*, central panels (b and e) compare *D. athabasca* and *D. lenape*, and right panels (c and f) compare *D. mahican* and *D. lenape*. Blue squares represent *D. athabasca* (WN), red circles represent *D. mahican* (EA), and orange triangles represent *D. lenape* (EB) focal populations in each plot. Top panels (a–c) show *Dxy*, and bottom panels (d–f) show *Fst*. Both measures of genetic divergence are averaged across all sequenced genes. Note that these relationships avoid the problem of nonindependent data points by averaging *Fst* or *Dxy* values between each focal population with all heterospecific populations. Error bars indicate 95% confidence intervals between the focal population and heterospecific populations. Only populations with greater than three individuals are considered (i.e., *D. mahican* QB and RIVR/LNOM and *D. lenape* LK populations were excluded; results did not change when these were included). *R* ^2^ values shown for each focal species (*D. lenape* trendlines in center panels are not shown due to very small range of geographical data points)

To more directly test for heterospecific gene flow between partially sympatric species, we asked whether genetic divergence is lower between sympatric heterospecific populations compared to increasingly distant allopatric populations. This approach is especially powerful with *D. athabasca* (WN) as it is the only species with significant genetic divergence between allopatric and sympatric populations (see Figure 4). However, we found no relationship between average genetic divergence and geographical distance between heterospecific populations within any species pair (Figure 8). The two partially sympatric species pairs showed the same level of average sequence divergence in sympatry versus distant allopatry (Figure 8). Results remained the same when only high *Fst* fragments were considered (*Fst* > 0.5) and when the comparison was made between pooled sympatric versus allopatric lines (data not shown). We also did not find evidence of heterospecific gene flow between *D. athabasca* and *D. mahican* using ABBA–BABA test (see Table S5).

As the above spatial analyses are likely to have low power due to few numbers of relevant SNPs and would detect gene flow only if it was substantial, we also tested the question using *IMa2* coalescent demographic simulations. Our results showed significant gene flow between *D. athabasca* and *D. mahican*. Estimated migration rates, per locus, per mutation, showed posterior probabilities with narrow unimodal peaks: From *D. athabasca* to *D. mahican*, the rate was 0.12 (m/μ; 1.9 × 10^−7^ per locus per generation), and from *D. mahican* to *D. athabasca*, the rate was 0.25 (m/μ; 3.9 × 10^−7^ per locus per generation) (Figure S2a). However, migration rates from *D. mahican* to *D. lenape* were not significantly different from zero, and the probability distribution of migration rates from *D. lenape* to *D. mahican* was relatively flat and bimodal, making it difficult to distinguish from incomplete lineage sorting (see Figure S2b).

### 3.5 Time of divergence and effective population size

Using *IMa2* simulations, we ran scenarios with three different neutral substitution rates, calibrated assuming three different divergence times between *D. affinis* and *D. athabasca* species complex: (1) 250 KY (so as to approach mutation rates assumed in Ford & Aquadro, 1996 and Wong‐Miller et al., 2017), (2) 1.48 MY (estimate based on Carson, 1976), and (3) 3 MY (estimate based on Bachenbach et al. 1996; Russo et al., 2013). Consistent with prior work, our results showed that divergence between *D. athabasca* and *D. mahican* was always older than between *D. mahican* and *D. lenape* (Table 1). Also, *D. athabasca* and *D mahican* consistently had larger effective population sizes than *D. lenape* (Table 1).

However, absolute estimates were at least an order of magnitude different between the three scenarios. Our first scenario estimates (assuming very high neutral substitution rates) did not significantly differ from those of Ford and Aquadro (1996) and Wong‐Miller et al. (2017), despite using different coalescent approaches (Table 1). In contrast, the second and third scenarios produced much older divergence times and substantially larger population sizes compared to all previous work (Table 1). Below, we argue that these older divergence times and larger population sizes are likely more realistic compared to previous estimates.

## 4 DISCUSSION

The present work characterized reproductive isolation (sexual isolation) and genomic isolation in the *D. athabasca* species complex. Our results indicate that the species complex is more reproductively isolated and substantially older than previously assumed.

### 4.1 Virtually complete sexual isolation between species pairs

First, our extensive behavioral mating assays revealed for the first time that sexual isolation is virtually complete between partially sympatric species pairs (*D. athabasca*–*D. mahican* and *D. mahican*–*D. lenape*) and is much stronger than previously assumed between the allopatric pair *D. athabasca* and *D. lenape*. These levels of sexual isolation are comparable to any taxonomically designated species pair of *Drosophila* (e.g., see Coyne & Orr, 1989; Yukilevich, 2012).

Moreover, between partially sympatric species pairs, there was no evidence of RCD, such that even distantly allopatric heterospecific populations (up to 4,000 km apart) showed complete sexual isolation. This result indicates that the mating preferences within each species are homogeneous (“species‐wide”) and have spread across all populations.

### 4.2 Random mating and near genetic panmixia within species

We also found that there is random mating between all conspecific populations within each species. This result is consistent with “species‐wide” sexual isolation and previous work showing that male courtship song (the target of divergent female mating preferences) is not significantly different between conspecifics of each species (Yukilevich et al., 2016). Within‐species random mating is also consistent with our results of: (1) no significant difference in nucleotide sequence and haplotype diversity across conspecific populations, (2) low absolute (*Dxy*) and relative (*Fst*) sequence divergence within each species, and (3) weak or no IBD across these wide‐ranging species.

In general, genetic divergence within species increased from *D. lenape* to *D. mahican* and to *D. athabasca*, which has twice the geographical range of the two eastern species. These patterns of within‐species divergence are similar to patterns found using allozymes and mtDNA sequence divergence (Johnson, 1978, 1985; Yoon & Aquadro, 1994). In total, all results imply high dispersibility and gene flow within each species and/or very recent population expansions (the latter possibility was not tested, but was suggested by negative Tajima's *D* values across the three species in the complex; data not shown). Based on *Fst* values, we estimate approximately 1.14 migrants per generation between furthest conspecific populations and 9.75 migrants per generation between closest populations (assuming evolutionary equilibrium).

### 4.3 Mixed evidence for heterospecific gene flow

The above near complete sexual isolation between partially sympatric species pairs suggests that heterospecific gene flow, if it occurs at all, should be very low. Previous work failed to find hybrids between *D. athabasca* (WN) and *D. mahican* (EA) in sympatry based on differences in chromosomal inversions (Miller & Voelker, 1969a, 1969b, 1972) and allozymes (Johnson, 1978). While Yoon and Aquadro (1994) found a shared mitochondrial haplotype between *D. mahican* (EA) and *D. lenape* (EB), they argued that this is likely due to shared ancestral polymorphism rather than gene flow as the haplotype was also found in allopatry. Recent whole‐genome analysis of Wong‐Miller et al. (2017) found mixed results: Based on several and mostly allopatric lines, their simulations indicated either very low or no gene flow.

In the present survey of 281 isofemales lines in allopatric and sympatric locations, at least 94% had a genetic composition of pure species. The remaining 6% showed weaker probability of species assignment, but based on their courtship song and sexual isolation values, they were found to be indistinguishable from pure species lines. More explicit tests failed to show evidence for substantial heterospecific gene flow in sympatry. Geographical regression analyses of heterospecific populations (i.e., relating genetic divergence to geographical distance) and ABBA–BABA test of introgression revealed that sympatric and distantly allopatric populations had the same levels of genetic divergence. However, these approaches are probably sensitive to the number of informative SNPs and, with our genetic resolution, could only detect heterospecific gene flow if the rates were substantial.

Thus, we also used coalescent demographic simulations. Our results did reveal significant gene flow, especially between *D. athabasca* and *D. mahican* (i.e., estimates of gene flow rates between *D. mahican* and *D. lenape* were either zero or unreliable). However, even between *D. athabasca* and *D. mahican*, the estimated overall genetic contribution of gene flow to each species was found to be similar to that of the neutral mutation rate per generation. So while neither of our approaches showed high level of heterospecific gene flow, our simulation results revealed that low gene flow likely occurred after initial divergence between these species.

### 4.4 Substantially older divergence times

Finally, we showed that the timing of divergence in this species complex is unlikely to be as recent as previously assumed. Ford and Aquadro (1996) and Wong‐Miller et al. (2017) estimated that *D. athabasca* (WN) diverged from eastern species only 23,000–35,000 years ago, and *D. mahican* (EA) and *D. lenape* (EB) diverged only 5,000–9,000 years ago. Such extremely recent divergence times were also attained in our study only if we assumed a very high neutral substitution rate, as was done in Wong‐Miller et al. (2017) based on Haag‐Liautard et al. (2007) study that did not isolate neutral mutation rate per se. However, this would force the timing of divergence between the *D. athabasca* species complex and *D. affinis* and *D. pseudoobscura* to be at least an order of magnitude younger than is generally accepted (Beckenbach et al., 1993; Carson, 1976; Russo et al., 2013; Tamura et al., 2004).

When a lower neutral mutation rate was assumed based on calibrated divergence time between Hawaiian *Drosophila* (Carson, 1976) and other phylogenetic studies (Beckenbach et al., 1993; Russo et al., 2013; Tamura et al., 2004), our simulations produced correspondingly much older divergence times in this complex; at a minimum 191,695 years ago between *D. athabasca* and eastern species, and 98,125 years ago between *D. mahican* and *D. lenape*.

These older divergence times are also supported by indirect evidence. First, crossing any *D. athabasca* species with *D. affinis* produces male and female hybrid sterility in at least one reciprocal cross (Miller, 1950). This observation is consistent with older calibrated divergence times between these species as postzygotic isolation appears roughly between 1 million‐year‐old *Drosophila* (Coyne & Orr, 1989, 1997). Second, all three species of the *D. athabasca* complex contain many fixed chromosomal inversion differences and have diverged in multiple phenotypic and behavioral traits (see above). It would be unprecedented that all of these genetic and phenotypic changes occurred in only a few thousand years.

### 4.5 Speciation in the *D. athabasca* species complex and relationship to enhanced isolation in sympatric *Drosophila*

Our study provides several insights about speciation in this species complex and its relationship to the broader pattern of enhanced sexual isolation in sympatric *Drosophila* and reinforcement (Coyne & Orr, 1989, 1997; Yukilevich 2012). By focusing on partially sympatric species pairs, we were able to test whether reinforcement explains rapid evolution of sexual isolation in this system. Our results of near complete sexual isolation and total lack of RCD in sympatric zones does not support reinforcement (Coyne & Orr, 2004; Servedio & Noor, 2003). Moreover, the fact that these species are interfertile also disfavors reinforcement in this system (Miller & Westphal, 1967; Hey, 1988; R. Yukilevich personal observation).

However, it may be premature to completely rule out some form of reinforcement selection. First, the detection of low heterospecific historical gene flow between *D. athabasca* and *D. mahican* could be consistent with a secondary contact scenario as theoretical models have shown that reinforcement is most likely when gene flow is not too high (see Coyne & Orr, 2004; Nosil, 2013; Servedio & Noor, 2003). Second, our results of random mating and rampant conspecific gene flow within each species may imply that the signature of RCD could have been erased in the past by homogenizing the differences between allopatric and sympatric populations. Third, while these species produce fertile and viable hybrids in the laboratory (R. Yukilevich personal observation), a detailed study of F1 and F2 hybrid fitness in an ecological and sexual context has yet to be done to ensure that there are no quantitative costs to hybridization. It is also unknown whether speciation occurred as a byproduct of RCD from other closely related neighboring species, such as *D. affinis* and *D. algonquin*, which also deserves further inquiry.

Alternative to reinforcement, we may envision either: (1) pure allopatric speciation or (2) primary contact parapatric/sympatric speciation. First, purely allopatric speciation in this system would be very surprising as there are presently no phylogenetically independent allopatric *Drosophila* pairs that show such high rates of sexual isolation as found in sympatric *Drosophila* (Coyne & Orr, 1989, 1997; Yukilevich, 2012). This suggests that sympatric conditions were necessary for rapid speciation in the *D. athabasca* species complex. Parapatric/sympatric speciation is plausible as the geographical ranges of these species were likely historically further south in the United States and more constrained during previous ice ages (Brubaker, Anderson, Edwards, & Lozhkin, 2005; Clark et al., 2009; Hultén, 1937; Pieloue, 1991). The discovery of *D. athabasca* and *D. mahican* syntopically residing in the Smoky Mountain National Park (Tennessee) is consistent with this scenario and suggests an ancestral region. Under this scenario, we would not expect a pattern of RCD if complete sexual isolation first evolved in parapatry/sympatry in the southern United States, and then, some populations became allopatric as they moved northwest after the ice age.

Additional work is necessary to further characterize the mechanisms of speciation in this system. This includes determining the fitness of hybrids in both laboratory and field conditions, studying the role of chromosomal inversions in this speciation event, and deciphering historical ranges and predicted range expansions using additional genetic analyses. This and other work are necessary to further test whether reinforcement or primary contact/parapatric speciation drives rapid speciation in nature.

## CONFLICT OF INTEREST

None declared.

## AUTHOR CONTRIBUTIONS

R. Yukilevich and L. Maroja conceived the study, performed experiments, analyzed the data, and wrote the manuscript. K. Nguyen, S. Hussain, and P. Kumaran performed experiments and analyzed the data.
