# Supplementary material for: Rapid sexual and genomic isolation in sympatric Drosophila without reproductive character displacement
Source: Ecol Evol. 2018 Feb 11;8(5):2852–67. doi: 10.1002/ece3.3893 (PMC5838044; doi:10.1002/ece3.3893)
Supplement: Supplementary file 1 [file ECE3-8-2852-s001.docx]

**D**

**A**

**B**

**C**

***athabasca***

***mahican***

***lenape***

***affinis***

***athabasca***

***mahican***

***lenape***

***athabasca***

***mahican***

***lenape***

***athabasca***

***mahican***

***lenape***

***affinis***

**Supl. Fig. S1.** Population genetic statistics across sequenced gene fragments within each species of *D. athabasca* complex and its sibling species *D. affinis.* Number of gene fragments studied is shown above each bar and error bars indicate 95% confidence intervals. **A**) Average nucleotide diversity per site (**π**) per gene fragment for autosome (left bars) and X-chromosome (right bars) (Kruskal-Wallis test (Auto. vs. X): *D. affinis*: Z-value = 1.35, *p* = 0.18; *D. athabasca*: Z-value = 2.31, *p* = 0.02; *D. mahican*: Z-value = 2.06, *p* = 0.039; *D. lenape*: Z-value = 2.53, *p* = 0.01; same results are seen for average number of nucleotide differences (*k*); data not shown). Measures of nucleotide diversity per chromosome decrease from chromosomes B > E > C > A > D, with B being significantly different from all others (data not shown). **B**) Nucleotide sequence diversity per site (**π**) across all gene fragments. Conspecific populations are not significantly different from each other (ANOVA analyses based on all gene fragments per population; not shown). Overall statistics between species with population means as replicates per species: *D. affinis* = 0.011 (n = 1), *D. athabasca* = 0.0063 (n = 6), *D. mahican* = 0.0068 (n = 10), *D. lenape* = 0.0055 (n = 4): ANOVA F-ratio = 24.7, *p* < 0.0001; posthoc Tukey’s test significant at *p* < 0.05 between *D. affinis* versus all three species of *athabasca* complex and between *D. lenape* versus *D.athabasca/D.mahican*. **C)** Haplotype diversity (*Hd*) across all gene fragments. Conspecific populations of each species are not significantly different from each other (ANOVA analyses based on all gene fragments per population; not shown). Overall statistics between species with population means as replicates per species: *D. athabasca* = 0.57 (n = 6), *D. mahican* = 0.66 (n = 10), *D. lenape* = 0.59 (n = 4): ANOVA F-ratio = 8.25, *p* < 0.0001; posthoc Tukey’s test significant at *p* < 0.05 between *D. mahican* versus *D. athabasca*. **D)** Average estimates of *Fay and Wu’s H* statistic across gene fragments on autosomes (left bars) and X-chromosome (right bars): Statistical tests for Auto. vs. X are not significant (ANOVA analyses not shown).
