# Supplementary material for: Rapid sexual and genomic isolation in sympatric Drosophila without reproductive character displacement
Source: Ecol Evol. 2018 Feb 11;8(5):2852–67. doi: 10.1002/ece3.3893 (PMC5838044; doi:10.1002/ece3.3893)
Supplement: Supplementary file 2 [file ECE3-8-2852-s002.docx]

**B**

**A**

**Supl. Fig. S2.** Estimated posterior probabilities of historical migration rates between sympatric species pairs: A) *D. athabasca* (WN) - *D. mahican* (EA), and B) *D. mahican* (EA) - *D. lenape* (EB) sympatric populations. Migration rate here is relative to neutral mutation rate (u = 5.12 x 10^-9^) per generation (M/u). Migration rates in terms of *2Nm* are qualitatively similar and all show peak probabilities << 1.0. Probabilities for different migration directions are shown in blue versus red lines for each species pair comparison. The probabilities are based on coalescent demographical simulations using IMa2 software (see text). Peak probabilities are indicated above each migration direction, with asterisks indicating statistical significance based on migration rate likelihood ratio test “LLR-test” (see text). NS = not significant.
