# Supplementary material for: Rapid sexual and genomic isolation in sympatric Drosophila without reproductive character displacement
Source: Ecol Evol. 2018 Feb 11;8(5):2852–67. doi: 10.1002/ece3.3893 (PMC5838044; doi:10.1002/ece3.3893)
Supplement: Supplementary file 7 [file ECE3-8-2852-s007.docx]

**Supl.Table S5.** ABBA-BABA test of gene flow between sympatric and allopatric populations of *D. athabasca* (WN) and *D. mahican* (EA).

**Notes:** Using the 4,236 variable sites, we identified bi-allelic sites that had derived alleles in either *D. athabasca* (WN) or *D. mahican* (EA) relative to outgroup *D. affinis* and were polymorphic between two conspecific sequences being compared (e.g. H1 = *D. mahican* allopatric, H2 = *D. mahican* sympatric, *D. athabasca*, *D. affinis*). We randomly picked two conspecific sequences (H1, H2) and for each sequence we determined the number of sites that matched the other species allele (ABBA or BABA). Statistic *D = (# ABBA sites – # BABA sites) / (# ABBA sites + # BABA sites)* (see ***Material and Methods*** for details). For each comparison type, average *D* statistic (shown above) was calculated and determined if significantly different from zero and across different comparisons using ANOVA. We separately tested for evidence of gene flow from *D. athabasca* to *D. mahican* and from *D. mahican* to *D. athabasca*. Three different comparisons are shown for each test: allopatric-allopatric sequences, sympatric-sympatric sequences, and allopatric-sympatric sequences.
