# Supplementary material for: Rapid sexual and genomic isolation in sympatric Drosophila without reproductive character displacement
Source: Ecol Evol. 2018 Feb 11;8(5):2852–67. doi: 10.1002/ece3.3893 (PMC5838044; doi:10.1002/ece3.3893)
Supplement: Supplementary file 8 [file ECE3-8-2852-s008.docx]

**Supl. Table S6.** Analyses of STRUCTURE replicates to determine the true number of groups “K” among the 281 analyzed individuals within the athabasca species complex.

Notes: Evanno et al. 2005 methods were used in analyses. True K is usually described as that having highest LN P(d) value and/or highest delta (CHANGE) L(K) value. See Evanno et al. 2005 for further description.
